# Supplementary material for: CMTR1 is recruited to transcription start sites and promotes ribosomal protein and histone gene expression in embryonic stem cells
Source: Nucleic Acids Res. 2022 Feb 25;50(5):2905–22. doi: 10.1093/nar/gkac122 (PMC8934662; doi:10.1093/nar/gkac122)
Supplement: gkac122_Supplemental_Files [file gkac122_supplemental_files.zip › Table S1 legends.docx]

**Table S1. Summary of ChIP-seq and RNA-seq analysis of individual genes** For CMTR1 siRNA RNA-seq, analysis from two siRNAs were shown, genes with significant changes in their transcript levels after CMTR1 siRNA knockdown were labelled (fold change ≥1.5 or ≤0.67, FDR adjusted P-value ≤ 0.05). Significantly down-regulated genes were labelled as -1, significantly up-regulated genes were labelled as 1, genes without significant changes were labelled as 0.

For CMTR1 and RNMT ChIP-seq, CMTR1 or RNMT-enriched genes were defined as those genes that had ChIP enrichment fold change ≥1.5, FDR adjusted P-value ≤ 0.05. For siCMTR1 RNAPII ChIP-seq, those genes with significant changes in their RNAPII levels were labelled (fold change ≥1.5 or ≤0.67, FDR adjusted P-value ≤ 0.05). Significantly down-regulated genes were labelled as -1, significantly up-regulated genes were labelled as 1, genes without significant changes were labelled as 0.
